# Diseases of the Nervous System

**Published:** 1906-03-24

**Authors:** 


### Progress in Medicine and Surgery.

### DISEASES OF THE NERVOUS SYSTEM.

(*Continued from page* 407.)

**Acute Anterior Poliomyelitis.**—While the anatomical conditions found in acute anterior poliomyelitis are definite and constant, consisting mainly in destruction and atrophy of the large motor cells of the anterior cornua, and of the corresponding nerve roots, and of more or less vascular change in the affected cord areas, the ætiology of the disease is obscure, and its pathology still somewhat uncertain. Much of the more recent literature on the subject has been summarised and examined by T. A. Hoch.[10] Age, season, trauma, and infectious diseases are the most important ætiological factors. Pre-eminently a disease of early childhood, the majority of cases occur at about three years or under, when the child is beginning to walk and the spinal centres are therefore unusually active, and consequently unusually vulnerable. The disease may, however, occur as late as sixty. It is most prevalent in the hot summer months, especially from June to September. Trauma, a very frequent antecedent in the minds of parents, has been denied much influence in medical text-books, yet several writers have cited instances of the association of the disease with injury. Thus Bullen, von Leyden, Stark, Burckhardt, and others have reported cases in which paralysis followed injury, and structural changes were found in the grey matter of the cord. Several of these patients were adults, however, and the course seems to have been chronic and progressive rather than acute. Burckhardt's patient was a boy aged one year, whose face was scalded with boiling water, and who in a few days developed vomiting, fever, and paralysis of the limbs and trunk. Death occurred in four days, and post-mortem macroscopical and microscopical changes were evident in the grey matter. The disease behaves like one of an infectious nature, and several epidemics have been recorded. One of the largest of these occurred in Vermont in the United States, where, during the summer of 1894, 119 cases developed, of whom 18 died. Other epidemics have been described in other parts of the States, in Stockholm, Italy, and various other places. Pasteur recorded the attack of a whole family of seven children within ten days, and many examples are quoted of its multiple incidence in the same family, house, or street. One observer, Johannessen, suggested a relationship between this disease and the gastro-intestinal disturbances of young children, and swollen mesenteric glands and hyperæmic Peyer's patches appear to have been found in some cases. Lepine observed it during the course of typhoid fever, and it has been found associated in the same epidemic with cases of neuritis. Diligent search has been made for bacteria in the cord and cerebro-spinal fluid without any uniform result. The disease appears, however, to be due to toxins of bacterial or other origin. Among the organisms which have been found in the cerebro-spinal fluid obtained by lumbar puncture may be mentioned staphylococcus pyogenes albus, the diplococcus of Sternberg—or a very similar organism, and the Weichselbaum-Jaeger meningococcus, besides other unnamed cocci. Acute anterior poliomyelitis has, moreover, been produced in rabbits by inoculation with splenic cultures from a patient dead of typhoid fever, but no bacilli could be isolated from the rabbits infected. In other cases the fluid has proved sterile. Clinically, where the symptoms have been occasionally typical of some other disease, such as transverse myelitis or Landry's paralysis, the anterior cornual changes typical of infantile paralysis have been found on examination. Clinically and anatomically, indeed, Landry's acute ascending paralysis and infantile paralysis sometimes overlap, but in the former signs of an acute infection may be present, such as enlargement of spleen and lymph glands and albuminuria. A hæmorrhagic poliomyelitis was found in one case by Bassœ. The pathology of infantile paralysis is also unsettled. We do not know why one particular portion of the cord is attacked, nor certainly whether the nervous elements or the vessels are primarily involved. Charcot, who examined long-standing cases in which inflammatory phenomena had long since subsided, regarded the disease as primarily affecting the nervous matter, and he is followed by some recent writers. The majority of modern writers, however, regard the atrophy of nerve tissue not as a primary condition, but as secondary to interstitial changes. Possibly both cells and vessels may be acted on simultaneously by the poison, but a study of early acute cases shows either a more or less widespread hæmorrhagic condition of the cord or a violent inflammation involving more particularly the vessels without visible structural change in the nerve cells. The vessels are dilated and the perivascular spaces enormously distended by cells, while there is hypertrophy and hyperplasia of the neuroglial cells. It seems certain that such pressure must exert a traumatic influence on the nerve cells, and the widespread scar tissue which is found replacing these suggests that they have been destroyed by pressure. Probably anterior poliomyelitis is the result of various poisons, and cases have been described as due to lead and arsenic poisoning. The undoubted effect of lead in producing distinct changes in the cells of the central nervous system has also been declared by many to be due to primary vascular changes and secondary cell destruction, and perivascular cell infiltration has been shown to exist in some cases of anterior poliomyelitis associated with plumbism. The selection of the anterior horns for the special stress of the poison may be partly explained by the anatomical conditions of their blood supply. The grey matter of the anterior horns receives terminal arteries which do not anastomose, consequently their circulation is poor and sluggish, and the occurrence of embolism and thrombosis, which have been repeatedly demonstrated in the anterior spinal arteries, is favoured. As Hoch[11] has shown in a fatal case of his own occurring in a youth of 15, it is not only the parts served by the anterior spinal arteries, however, which are involved, the peripheral vessels may also be affected, and the parts supplied by them show, as in his case, anæmic necrosis, small hæmorrhages, and cell destruction. The perivascular infiltration is shown in these sections to be confined to the grey matter, and rarely to occur in the white. The distribution of the lesions in this case also seems to show that the grey matter of the cord receives rather more nutrition from the peripheral vessels than is generally taught, especially in the antero-lateral regions. Reference has already been made to the view expressed by Hoch that sometimes a relationship exists between acute anterior poliomyelitis and Landry's paralysis. Three interesting cases of the latter disease may therefore be referred to here,[12] two of whom recovered. One, a man of 24, who had suffered from gonorrhœa and articular rheumatism, after a few days' malaise and fever developed numbness and tingling of the legs, followed by a rapidly ascending paralysis, which involved all the limb and trunk muscles, and also those of speech and swallowing, and the sphincters. The patient appeared on the point of death from asphyxia, but then began slowly to improve, and six weeks or so later was only paralysed in the lower limbs. Fever, enlargement of the spleen and lymphatic glands, and albuminuria were other features observed. The second case was that of a healthy boy of 17, who felt tired one day, and then rapidly developed almost complete paralysis of the trunk and limbs, without atrophy, sensory disturbance, other than tenderness on suppression of the calf muscles, or constitutional symptoms. Complete recovery occurred in three months. The third case, also in a young man, had a similar history to the second, but death occurred on the seventh day. This patient had, however, suffered twenty months previously from a similar but less severe attack, from which recovery was perfect. Only in the first case was a slight atrophy of certain muscles observed, and, as is usual in Landry's paralysis, reaction of degeneration was absent. While the pathology of this disease is still obscure, the absence of muscular atrophy and of electrical degeneration must distinguish most cases from anterior poliomyelitis of the ordinary acute type.

[10] Jour. Nerv. and Ment. Dis., Oct. [11] Ibid., Sept. [12] Glasg. Med. Jour., Aug.

